# Editors' Note

**DOI:** 10.5195/ijt.2020.6317

**Published:** 2020-06-30

**Authors:** Ellen R. Cohn, Jana Cason

The *International Journal of Telerehabilitation* (IJT) is a biannual journal dedicated to advancing telerehabilitation by disseminating information about current research and practices. The journal is indexed by PubMed and Scopus.

## ISSUE OVERVIEW

The current issue of the multi-disciplinary *International Journal of Telerehabilitation* (IJT) features two impactful articles about telerehabilitation in countries that are ‘oceans away' from the IJT virtual offices. One article describes the status of speech-language pathology clinical services in the Maldives, and the other, the management of Spinal Cord Injury (SCI) in the United Kingdom. Both publications propose how telerehabilitation can be employed to deliver greater access to services in underserved regions.

The content of a third article is unique for the *International Journal of Telerehabilitation* (IJT). It presents a case study in which an astute physical therapist referred a patient to a neurologist. Beyond the relative rarity of the medical issue (i.e., felcine meningioma) the circumstances are unusual because it was a telephone conversation that precipitated the life-saving referral to a neurologist.

Telephone audio-based communication is not often reported in journals as a telehealth option. Why not? That some state boards require that telerehabilitation be conducted via videoconferencing with audio and video likely discourages telephone use. (Clinicians, of course, must employ appropriate telecommunication technologies to diagnose, refer, and treat clients.) And via pictorial representations, telehealth has been “branded” by professional associations and their industry partners as computer or tablet based. Yet, telephone communication is consistent with the American Physical Therapy Association's definition of telehealth as the use of electronic communication to remotely provide health care information and services (American Physical Therapy Association, 2019). And to more broadly contextualize the importance of telephone communication in the United States, each year an estimated 240 million calls are made to 9-1-1.^1^ Emergency medical circumstances are initially triaged and even successfully managed (e.g., choking, delivery of babies) via telephone conversations that are audio-only. Telephone calls are also routinely employed by medical practices, pharmacies, poison control centers, and suicide prevention hotlines. Many medical offices hold early morning phone consultations to receive unscheduled calls from patients or caregivers. These conversations are used to issue medical advice, to schedule the patients who need to be seen in the office that day on an urgent basis, and to triage the patients who must be referred with haste to the nearest hospital emergency department. Considering the definitional inclusiveness (i.e., telephone employs telecommunication), and overwhelming precedent, it seems ill-conceived to summarily fail to value appropriate telephone based telerehabilitation.

It is also important to recognize that the prohibition or “writing off” of all telephone-based telerehabilitation fails to acknowledge the social and economic disparities that impede access to healthcare, worldwide. Vulnerable populations may not have the access or training to use the internet, smart phones, tablets, or computer-based videoconferencing.

There is unfortunately a paucity of research to compare the efficacy of audio-only and audio-visual telehealth in circumstances that might be well served by telephone communication. Hybrid approaches to telerehabilitation that employ both in-person and treatment via videoconferencing also require further research. A fourth article describes the combined effects of telehealth and modified constraint-induced movement therapy for individuals with chronic hemiparesis.

A fifth article in the current issue reports that “perceived usefulness” may be an important factor associated with health care providers' intent to use telerehabilitation for pulmonary rehabilitation. Indeed, “perceived usefulness” is currently supremely relevant in motivating many aspects of impactful human behavior. Perceived usefulness and potential harm have and will influence medication/vaccine adherence. In the current COVID 19 pandemic environment, perceived usefulness is central to the willingness of citizens to voluntarily abide by social distancing, and to wear protective face covering (i.e., masks) in public. It is critical to consider key indicators of perceived usefulness when developing telerehabilitation programs.

## INTERNATIONAL READERSHIP

This issue is the first issue of the 12^th^ year of IJT publication. Between May 1, 2019 - May 1, 2020 there were 19,010 new users from a total of 130 countries. As per 2020 Google Analytics, 25 countries with the highest readership (in descending order) were: United States, Australia, Canada, United Kingdom, Philippines, Germany, Singapore, Italy, South Korea, Brazil, Hong Kong, Portugal, Ireland, Spain, Chile, Iran, Turkey, France, Finland, Columbia, Netherlands, Saudi Arabia, and Austria. All continents except for Antarctica are represented in the IJT readership.

## Footnotes

https://www.nena.org/page/911Statistics

## CALL FOR SUBMISSIONS

We cordially invite submissions to the Fall 2020 issue by mid-August 2020. IJT accepts original research, case studies, viewpoints, technology reviews, book reviews, and country reports that detail the status of telerehabilitation.

Sincerely,

Ellen R. Cohn, PhD, CCC-SLP, ASHA-F

IJT Editor

Jana Cason, DHSc, OTR/L, FAOTA

Senior Associate Editor

